# Program design features that can improve participation in health education interventions

**DOI:** 10.1186/1471-2288-7-47

**Published:** 2007-11-09

**Authors:** Enza Gucciardi, Jill I Cameron, Chen Di Liao, Alison Palmer, Donna E Stewart

**Affiliations:** 1 School of Nutrition Ryerson University, Toronto, Ontario, Canada; 2 University Health Network Women's Health Program, Toronto, Ontario, Canada; 3 Department of Occupational Science and Occupational Therapy, University of Toronto, Ontario, Canada; 4 Toronto Rehabilitation Institute, Toronto, Ontario, Canada; 5 Department of Laboratory Medicine and Pathobiology, University of Toronto, Ontario, Canada; 6 Department of Family and Community Medicine at the University of Toronto, Ontario, Canada; 7 Faculty of Medicine, University of Toronto, Ontario, Canada

## Abstract

**Background:**

Although there have been reported benefits of health education interventions across various health issues, the key to program effectiveness is participation and retention. Unfortunately, not everyone is willing to participate in health interventions upon invitation. In fact, health education interventions are vulnerable to low participation rates. The objective of this study was to identify design features that may increase participation in health education interventions and evaluation surveys, and to maximize recruitment and retention efforts in a general ambulatory population.

**Methods:**

A cross-sectional questionnaire was administered to 175 individuals in waiting rooms of two hospitals diagnostic centres in Toronto, Canada. Subjects were asked about their willingness to participate, in principle, and the extent of their participation (frequency and duration) in health education interventions under various settings and in intervention evaluation surveys using various survey methods.

**Results:**

The majority of respondents preferred to participate in one 30–60 minutes education intervention session a year, in hospital either with a group or one-on-one with an educator. Also, the majority of respondents preferred to spend 20–30 minutes each time, completing one to two evaluation surveys per year in hospital or by mail.

**Conclusion:**

When designing interventions and their evaluation surveys, it is important to consider the preferences for setting, length of participation and survey method of your target population, in order to maximize recruitment and retention efforts. Study respondents preferred short and convenient health education interventions and surveys. Therefore, brevity, convenience and choice appear to be important when designing education interventions and evaluation surveys from the perspective of our target population.

## Background

Due to the growing availability of medical and health information through various sources and forms of mass media [1], more and more people are becoming consumers of health information [2]. However, this accumulation of knowledge does not necessarily result in adequate information about one's health or illness. In fact, the public health literature suggests that a large percentage of individuals are unaware of the symptoms, mechanism and management of their illness, and many feel that their level of knowledge is unsatisfactory [3]. Therefore, delivering systematic, comprehensive and reliable information about the nature, prevention, detection and self-management of illnesses is imperative.

Governments and health professionals are recognizing this gap in knowledge, and increasing amounts of money and time have consequently been spent on designing and developing health education interventions. Health education interventions usually provide individuals with the necessary knowledge and/or skills regarding the nature of an illness; its mechanism, signs or symptoms; consequences of the illness; prevention, detection techniques or self-monitoring practices; and appropriate self-care, management and treatment methods. For instance, self-management education for chronic illnesses have been developed to empower participants, teach them skills and techniques, and improve their interaction with the healthcare systems to enhance the management of their chronic condition [4]. Interventions can either target specific groups of individuals such as individuals with specific illnesses; caregivers, family, and friends of those with illnesses; healthcare professionals; or the general public.

Interventions are usually evaluated by surveying or interviewing participants. Information such as their experiences, acceptability of the program, and relevant information about individuals before and after the completion of the education sessions are usually collected and analyzed. Evaluation or follow-up surveys provide important data regarding the effectiveness and limitations of the interventions.

In addition to the reported benefits of health education interventions across various health issues [5-10], the key to program effectiveness is participation and retention [11-15]. Unfortunately, not everyone is willing to participate in health interventions and its evaluation upon invitation. In fact, health education interventions are vulnerable to low participation rates [12,16-18]. Low participation not only limits these interventions from being effective and reaching their target population and goals, but also impedes the gathering of information necessary to further improve these programs. The amount of research on this issue is limited, and even fewer investigations look at the factors that could increase participation. We believe that intervention factors such as convenience and accessibility [19], length and frequency, and the characteristics of the setting or organization offering the intervention [20] can offset the perceived benefits and decrease participation of health education interventions [21].

The purpose of this study was to identify the preferred design features of health education interventions and intervention evaluation surveys in a general ambulatory population. By determining the extent to which people are willing to participate in education interventions based on various design features, program managers, educators and researchers can develop education initiatives that are more appealing, convenient and accessible to their population of interest, while at the same time increase program participation.

## Methods

### Subjects

Hospital outpatients were recruited during the spring of 2002 to complete a cross-sectional questionnaire while waiting for general diagnostic testing at the University Health Network, a consortium of three hospitals, in Toronto, Ontario, Canada. All participants were able to read and write in English and were willing to provide written informed consent. The institution's research ethics board approved the study.

### Measures

A questionnaire regarding the participation in health education interventions and intervention evaluation surveys was developed. A health education session was defined as an intervention that provides individuals with information about preventative health practices and/or disease self-management care. An evaluation survey was defined as a tool to assess issues related to the program and to one's health, such as physical, emotional or social well-being. The survey instrument was tested with ten outpatients and their family members for clarity and ease of completion prior to the commencement of the study. All individuals waiting in the diagnostic laboratory testing centres were approached by a research assistant and asked to participate in the study. After signing an informed consent, a questionnaire was given to participants to complete on their own.

**Figure 1:**
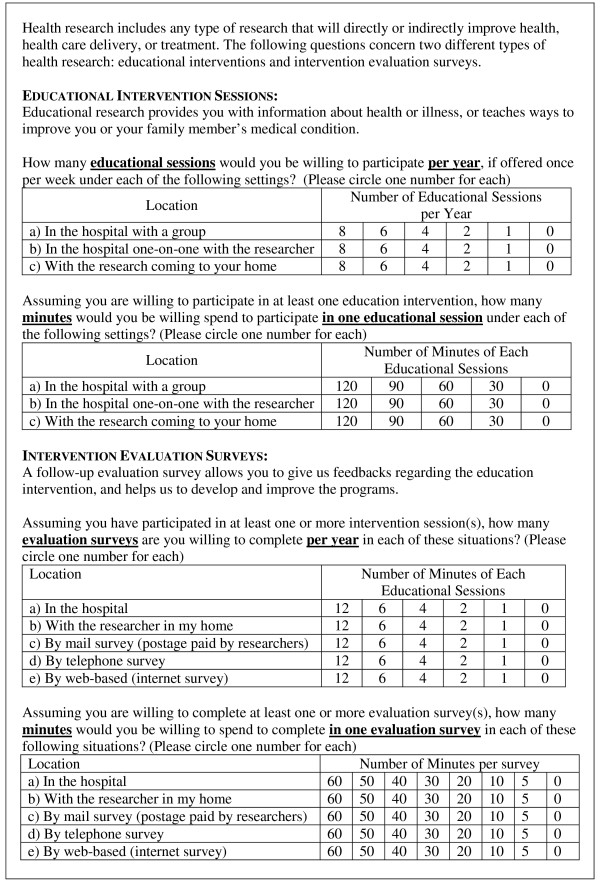
Extract of questionnaire.

The survey took 10–15 minutes to complete. Participants responded to a set of options concerning their participation in health education interventions and intervention evaluation surveys, in principle (See Figure 1 for an extract of the questionnaire). For each of the three settings (i.e., in hospital within a group, in hospital one-on-one with an educator and in the home with an educator), participants were asked to circle the number of health education intervention sessions (0, 1, 2, 4, 6, 8 sessions) they were willing to attend in a year if offered at a frequency of once per week, and the amount of time (0, 30, 60, 90, 120 minutes) they were willing to spend in an education session assuming they would have completed at least one education session. For each of the five survey methods (i.e., in hospital, in home with an interviewer, by mail, via phone and over the Internet), participants were asked to circle the number of evaluation surveys (0, 1, 2, 4, 6, 12 surveys) they were willing to complete in a year, assuming that they attend at least one intervention session, and the amount of time (0, 5, 10, 20, 30, 40, 50, 60 minutes) they were willing to spend completing a survey assuming they would have completed at least one evaluation survey.

### Statistical Analysis

Column bar graphs were used to summarize respondents' willingness to participate in health education interventions and evaluation surveys, based on their choices for the participation length and frequency under various settings and survey methods. While the education setting or survey method categories were represented by different shadings, the length and frequency choice categories were also collapsed to make interpretations of graphs easier. The two-way Chi-square tests were used to assess the significant difference in the distribution of the frequency and length the participants willing to spend in an intervention session or an evaluation survey, according to setting or survey methods, respectively. For all analyses, results were considered statistically significant where p-value <0.05. The data was analyzed using the Statistical Package for Social Sciences (version 11.5, SPSS Inc.) and/or Microsoft Excel (versions 2002, SP3, Microsoft Corp.)

## Results

**Figure 2:**
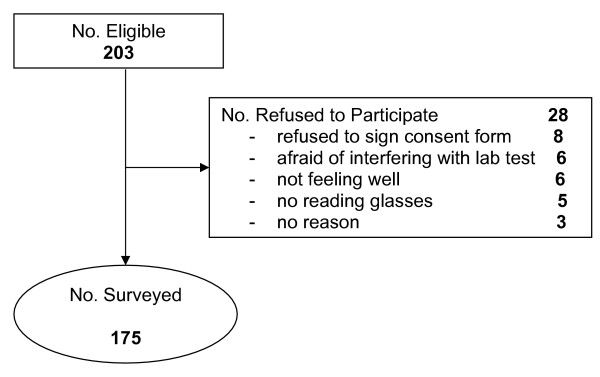
Participant recruitment procedure.

Two hundred and three eligible people were approached to participate in the study, of which 175 (response rate 86%) completed the survey. The common reasons for declining to participate in the study were: not feeling well (3%); not having their eye glasses (2%); worry that participating would interfere with their appointment (3%); and refusing to sign the consent form (4%), despite reassurance that their signature would not be linked with their responses (See Figure 2).

Our sample population had an equitable proportion of males (51.5%) and females, and the mean age was 44.5 (SD 13.8) with a range from 13 to 80 years of age. The majority of participants was born in Canada (66.9%), lived in the Greater Toronto Region (70.1%), primary language was English (92.2%), had some or a college/university degree (57.7%), was employed (67.7%) and had an annual income of or more than $40,000 (73.2%). Overall, 57.4% of our study participants had previously participated in health research, and 77.5% of participants indicated that their self-reported health status to be good to excellent. The characteristics of the study population are presented in Table 1.

**Table 1:** Characteristics of the study participants

*Continuous Variable*	*Mean*	*SD*^*a*^
Age (year)	44.5	13.8

*Categorical Variable*	*Frequency (n)*	*%*

Sex		
Female	79	48.5
Male	84	51.5
Education		
≤ High school	32	20.5
Some or college/university degree	90	57.7
Professional/graduate degree	34	21.8
Income		
≤ $40 000	38	26.8
> $40 000	104	73.2
Working Status		
Working for pay	107	67.7
Not working for pay	51	32.3
City of residence		
Greater Metropolitan Area	89	70.1
Other	38	29.9
Place of birth		
Canada	105	66.9
Other	52	33.1
Previous research participation		
Yes	89	57.4
No	66	42.6
Internet access		
Yes	123	76.4
No	38	23.6
General health rating		
Poor	8	5.0
Fair	28	17.5
Good	67	41.9
Very Good	40	25.0
Excellent	17	10.6

^a ^± standard deviation (SD)

### Health Education Interventions

**Figure 3:**
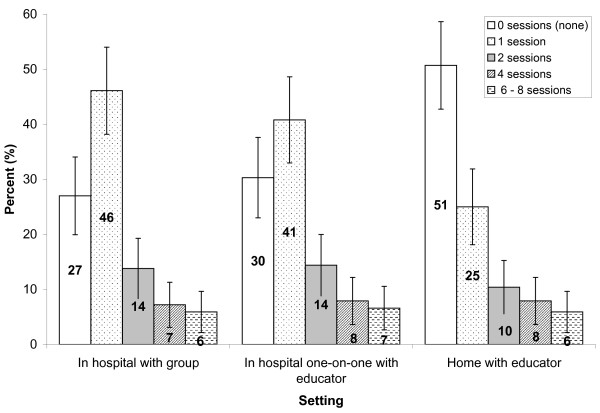
**Number of health education sessions respondents are willing to attend in a year by setting**. (All bars of the same education setting categories shading add up to 100%; 95% confidence intervals are indicated by the lines error bars).

In-hospital setting with a group was preferred by most participants, followed by one-on-one with an educator in-hospital, while about half of the respondents (50.7%, CI: 42.8, 58.7) disliked the idea of having an educator coming to their home. If health education intervention sessions were offered once per week over a one-year period, most respondents were willing to participate in only one education session regardless of the setting: 46.1% (CI: 38.2, 54.0) for in-hospital with a group, 40.8% (CI: 33.0, 48.6) for one-on-one in hospital with an educator, and 25% (CI: 18.1, 31.9) for in-home education sessions. As the number of sessions increased in a year, fewer respondents were willing to participate and smaller differences were observed among the three settings (Figure 3). Chi-square test indicated that the distribution of the preferred number of intervention sessions is significantly different between the settings (χ^2 ^= 24.882, p = 0.002, df = 8).

**Figure 4:**
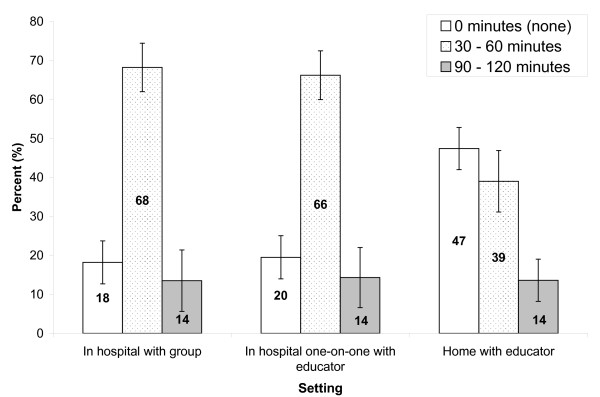
**Number of minutes respondents are willing to spend in a health education session by setting**. (All bars of the same education setting categories add up to 100%; 95% confidence intervals are indicated by the error bars).

Of the respondents who were willing to participate in education sessions, most people found 30 to 60 minutes per education session acceptable in-hospital setting, where 68.2% (CI: 60.7, 75.7) of respondents were willing to spend the noted amount of time in hospital with a group, and two-thirds of respondents (66.2%, CI: 58.7, 73.7) were open to one-on-one with an educator in hospital. Although nearly half of the participants (47.4%, CI: 39.5, 55.3) were reluctant to spend any time in an education session with an educator coming to their home, 39.0% were willing to participate for 30–60 minutes by this format. A small, yet similar proportion of respondents (14%) were willing to spend between 90 and 120 minutes in an education session across all three settings (see Figure 4 and additional file 1). Again, Chi-square tests showed that the distribution of the preferred length of each intervention sessions to be statistically different among the three setting (χ^2 ^= 43.274, p < 0.001, df = 4).

### Intervention Evaluation Surveys

**Figure 5:**
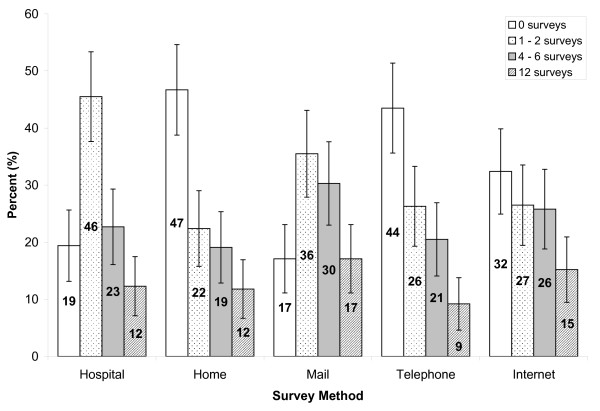
**Number of health evaluation surveys respondents are willing to complete in a year by survey method**. (All bars of the same survey method categories add up to 100%; 95% confidence intervals are indicated by the error bars).

The two most popular survey methods for evaluating the intervention were by participating in the hospital and by mail, while many were unwilling to complete any surveys at their home with the interviewer (46.7%, CI: 38.8, 54.6), by telephone (43.4%, CI: 35.5, 51.3), or over the Internet (33%, CI: 25, 40) (Figure 5). Of those willing to participate, the preference for the number of evaluation surveys willing to complete in a year was between one and two, with the leading response of 45.5% (CI: 37.6, 53.4) for the in-hospital method and 35.5% (CI 27.9, 43.1) by mail. As the number of surveys per year increased, a preference for mail and Internet responses was observed; yet the willingness to participate decreased overall. Chi-square test revealed significantly different distributions in the number of surveys participants are willing to take part in among survey formats (χ^2 ^= 61.475, p < 0.001, df = 12).

**Figure 6:**
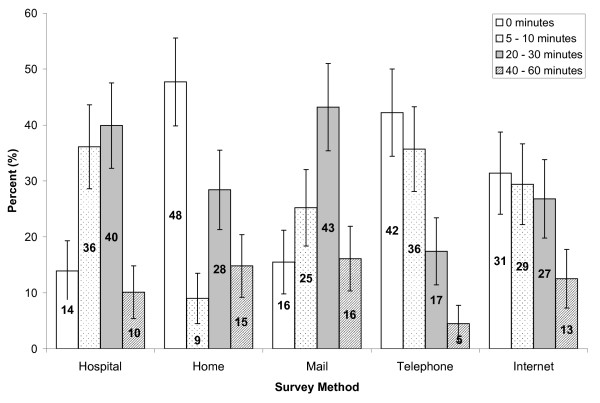
**Number of minutes respondents are willing to spend completing a health evaluation survey by survey method**. (All bars of the same survey method categories add up to 100%; 95% confidence intervals are indicated by the error bars).

For the two most popular survey methods, by mail and in hospital, 43.2% (CI: 35.4, 51.0) and 39.9% (CI: 32.3, 47.5) of respondents, respectively, were willing to spend 20 to 30 minutes filling out an evaluation survey (Figure 6). Although completing surveys by telephone was shunned overall, more than one third of the respondents (35.7%, CI: 28.1, 43.3) were willing to spend 5 to 10 minutes for a survey by this method. However, as the length of time increased, less people were willing to participate overall. Chi-square distributions of the preferred number of minutes willing to participate in evaluation surveys were significantly different across survey methods (χ^2 ^= 65.280, p < 0.001, df = 12).

## Discussion

Given that many health education interventions are provided in ambulatory settings, the goal of this study was to inquire about preferences regarding settings, survey methods, duration and frequency for interventions and evaluation survey from an ambulatory-based population. Our study findings suggest that research settings do influence people's willingness to participate in a health education intervention. Most individuals are willing to participate in only one education session per year for 30 to 60 minutes, preferably in hospitals, either in a group or one-on-one format. In addition to already being at the hospital for other reasons, hospital settings may be more comforting to individuals due to the presence of other people, or they may feel that hospital settings legitimize the proposed intervention or survey [20]. In fact, individuals may expect to receive some education about their illness while they are in the hospital. Also, individuals with chronic illnesses often feel isolated in their management of the disease [22], and family members may feel powerless in their support due to shortage of knowledge regarding the experience. In such cases, a group environment may provide participants with an opportunity to interact with others who have similar illnesses or concerns, resulting in a slightly higher popularity for group sessions over the one-on-one in-hospital format. However, some participants may dislike a group format for various reasons [21] and may want more interaction with knowledgeable health professionals [23], thus preferring a one-on-one setting. Providing individuals with a choice between individual and group education is ideal, but not always feasible, particularly when working with limited resources.

The majority of respondents are not willing to participate in education sessions in their home, which showed a different pattern from the responses towards in-hospital settings, possibly because this format may represent an invasion of their privacy and security. Certain individuals may also be discouraged from participating in home interviews due to fear of strangers, violence or abuse [24]. However, people who are financially or physically unable to travel to the location where a health education intervention is being offered [13,25-27], or who simply dislikes hospital settings [19], may prefer in-home education sessions or sessions in local community settings.

The type of survey methods used also influence how often and how long respondents are willing to spend completing an evaluation survey. In general, most people are willing to spend 20 to 30 minutes completing one to two surveys in hospital or by mail, but lower enthusiasm was observed for in-home or telephone methods. Surveys at hospitals can be completed while waiting for a medical appointment or immediately after the intervention, and mail surveys may be more popular because they allow participants to complete the survey at their convenience. Reasons for lower willingness to complete in-home surveys may be similar to those previously noted for in-home education sessions. Since telephone surveys usually require immediate completion, devoting a continuous period of time may be tiresome to some unless one is very motivated.

The preference to participate in studies by mail, as opposed to at home with an interviewer or by telephone, is problematic for program managers and researchers because mail surveys tend to have low response rates [28]. In addition, individuals with less education tend to be underrepresented in mail surveys, which may introduce a non-response bias [29]. Furthermore, people may forget or may feel less obliged to fill out mail surveys compared to other, more interactive modalities. However, mail surveys may provide greater and more accurate data when sensitive information is being sought, given the lack of an interviewer [24,30]. They are also more practical and inexpensive, costing much less per person than telephone or in-person interviews [30-32]. Several combined approaches can be used to enhance response rates from mail surveys, such as sending a reminder to participants within a month [28], re-mailing the survey [33,34], or including small monetary incentives [33-37]. In any case, using both mail and a subsequent telephone follow-up, with the option of completing the survey by telephone, may be most effective [38].

Currently, the Internet is not universally accessible and not all users are proficient at it [39], which may explain its unpopularity relative to hospital and mail surveys. Younger age and higher level of education is identified as the strongest predictors of Internet access and use [40]. Although most respondents had an education level of more than high school, the mean age of our sample population was 44.5, with 83.2% of respondents being over 30 years of age. This middle-aged population may be less receptive to computer technology and less likely to be Internet-orientated compared to a younger 18–29 age group [40], and thus might also account for the relative low preference for surveys via the Internet. However, as people become more Internet-orientated, this method may become more popular and practical [1]. Moreover, recent studies have shown an increasing preference for computer-based surveys because they offer more privacy and are easy to use [39,41]. One study showed that although e-surveys resulted in a lower response rate than postal surveys, the data quality was equivalent and was obtained in a shorter average response time, indicating that e-surveys could be a more feasible evaluation method in the future [42]. There is also exploration into the development of electronic and Internet-based health education intervention or support programs, which are getting favorable responses among participants in other research studies [43].

Due to the overall shortage of information regarding whether questionnaire length affects the response rate, this factor is still being debated among the academic literature. Some studies show that the response rate and the number of missing responses in a questionnaire are not related to questionnaire length, provided the questionnaire is well designed and easy to complete [28,31,44]. Other studies show that increasing the length of a questionnaire results in an increased burden on completion and a decreased response rate [45-48]. In our study, the number of respondents who were willing to participate in education intervention or evaluation surveys differed greatly between settings, especially at a lower frequency or for a shorter time-duration. Nevertheless, the majority of the respondents were agreeable to spend 30–60 minutes participating in an education session and 20–30 minutes completing a survey; however, as the number and length of education sessions and evaluation surveys increased, less people were willing to participate and less of a preference for any particular setting or survey method was observed. This inverse relationship demonstrates that most respondents are only willing to allocate up to a limited time when participating in education sessions and surveys. Individuals who are willing to participate in many education sessions or surveys, and for longer durations, may be motivated by different factors, and thus the location or method of the intervention or evaluation surveys may not be an issue. Consequently, designing short and convenient education programs and evaluation surveys appears to be important. For instance, an inverse relationship was found between program intensity and retention in health promotion work-site programs [11]; suggesting that intensive programs that demand more time, effort, and commitment may generate substantially lower participation and retention rates [49]. Furthermore, overly structured or inflexible interventions may not be optimal [50] and providing participants with options to choose from various settings or delivery methods that can easily fit into their schedules are likely more effective in retaining patients. Short and convenient scheduling of intervention sessions may be a key element in increasing participation, especially among full-time working individuals [11].

There are a few limitations associated with this study. Firstly, our study participants were recruited from hospital diagnostic testing centres and may not represent the general population. Our study participants may frequent the hospital more often than the general population, particularly since the majority of participants favoured a hospital-based setting for both the intervention and the evaluation survey. These findings may also imply a potential selection bias given the study population and setting were ambulatory-based. Nevertheless, this was our targeted population and the goal of this study was to identify their preferences. Furthermore, a normal distribution was observed among participants' self-reported health status, where 77 percent reported being in good to excellent condition, which is similar to the self-reported health status distribution observed among the general populations of Canadians, where 66 percent reported being in very good to excellent condition [51]. Our sample may not represent the non-English speaking population, who may adhere to different cultural beliefs that could influence one's motivation and decision to participate in education programs [52]. Yet, 33 percent of our study sample was born outside of Canada, and represented various non-western ethnic minority backgrounds including African, Asian, South American and East Europeans, thereby representing the opinions of a diverse and multicultural population. Lastly, this survey measured respondents' intentions to participate in a health education intervention and an evaluation survey based on various research design features; these intentions may not translate into actual participation if respondents were presented with the opportunity to take part in an education intervention or survey [53,54]. Nevertheless, our study findings capture a legitimate perspective (i.e., potential participants) on how to design better health education interventions and evaluation surveys to meet a population's preference.

## Conclusion

Our findings have important implications for the design and evaluation of education interventions. Our participants strongly preferred to receive educational interventions in a hospital setting either within a group or one-on-one with an educator. This was somewhat of a surprise at a time where the emphasis of healthcare is shifting towards a more home-based environment. This lack of interest in home-based interventions is also apparent in home-based survey evaluations. A small percentage of participants were interested in telephone or internet-based surveys and even fewer wanted interviewers coming into their homes.

In terms of duration and frequency for both health interventions and their evaluation, participants preferred short and convenient education sessions and evaluation surveys. Education interventions should not be too time intensive that people cannot fit them into their daily schedules. Therefore, balance is needed between the length of time necessary to deliver essential information and/or facilitation of new skills and the convenience and brevity participants desire to better design effective health education interventions. Furthermore, providing individuals with choice in regard to location and modalities may maximize recruitment and retention efforts. By considering potential participants' preferences in the design and implementation of health education interventions, it may be possible to improve recruitment efforts and increase participation rates.

## Competing interests

The author(s) declare that they have no competing interests.

## Authors' contributions

EG was involved in the conception and design of the research idea, coordinated the acquisition of the data, analyzed and interpreted the data, and drafted, reviewed and revised the manuscript. CDL analyzed and interpreted the data, draft, reviewed and revised the manuscript. AP was involved with the conception and design of the research idea, acquisition of the data, analyzed and interpreted the data, and drafted and reviewed the final manuscript. JC was involved with the conception and design of the research idea, coordinated the acquisition of the data, provided guidance with the analyses and interpretation of the data, and reviewed the manuscript. All authors read and approved the final manuscript.

## Pre-publication history

The pre-publication history for this paper can be accessed here:



## Supplementary Material

Additional file 1 Frequency table for the number of minutes respondents are willing to spend in a health education session by setting. The data provided present the frequency of respondents' choice of the 5 number of minutes they are willing to spend in a health education session by setting. Click here for file
