# Supplementary material for: Program design features that can improve participation in health education interventions
Source: BMC Med Res Methodol. 2007 Nov 9;7:47. doi: 10.1186/1471-2288-7-47 (PMC2204023; doi:10.1186/1471-2288-7-47)
Supplement: Additional file 1 — Frequency table for the number of minutes respondents are willing to spend in a health education session by setting. The data provided present the frequency of respondents' choice of the 5 number of minutes they are willing to spend in a health education session by setting. [file 1471-2288-7-47-S1.doc]

**Additional file 1**

Frequency data table for the number of minutes respondents are willing to spend in a health education session by setting

|  | In hospital with group | In hospital one-on-one with educator | Home with educator |
| --- | --- | --- | --- |
| 0 minutes (none) | 27 | 30 | 73 |
| 30 minutes | 55 | 56 | 23 |
| 60 minutes | 46 | 46 | 37 |
| 90 minutes | 5 | 3 | 4 |
| 120 minutes | 15 | 19 | 17 |
| Total | 148 | 154 | 154 |

The 5 categories were collapsed into 3 categories including “0 minutes”, “30-60 minutes” and “90-120 minutes” during data analysis, for what we believe to be the natural break points and reasonable lengths of education intervention sessions. Also, because of the low cell counts for the 90 minutes category we decided it would be best to collapsed it with the 120 minute category.
